# Livin promotes Th2-type immune response in airway allergic diseases

**DOI:** 10.1007/s12026-022-09294-9

**Published:** 2022-06-18

**Authors:** Yue Wang, Zhiyu Xiang, Miaomiao An, Huijing Jia, Chunyan Bu, Yanfeng Xue, Yao Wei, Ruiying Li, Xueping Qi, Fengli Cheng, Changqing Zhao, Jinmei Xue, Pingchang Yang

**Affiliations:** 1 Department of Otolaryngology, Head & Neck Surgery, The Second Clinical Medical College, Shanxi Medical University, Taiyuan, China; 2 Department of Otolaryngology, Head & Neck Surgery, The First Hospital of Yulin, Yulin, China; 3 Special Needs Ward, Shanxi Cancer Hospital, Taiyuan, China; 4 Department of Otolaryngology, Head & Neck Surgery, The Second Hospital of Shanxi Medical University, Taiyuan, China; 5 Key Research Laboratory of Airway Neuroimmunology, Shanxi Province, Taiyuan, China; 6 Research Center of Allergy & Immunology, Shenzhen University School of Medicine, Shenzhen, China

**Keywords:** AAD, Livin, GATA3, CD4^+^ T cells, Th2 differentiation

## Abstract

**Objectives:**

To investigate the effects of livin on the Th2 immune response in airway allergic diseases (AAD) and explore the interaction among livin, GATA3, IL-4 in peripheral blood CD4^+^ T cells of AAD patients.

**Methods:**

WT mice and livin KO mice were developed for model of AAD. Th2 cell levels in the lung tissues and spleen were assessed by flow cytometry. Also, it was assessed in the culture after exposing to livin inhibitor (Lp-15); the protein and mRNA levels of livin, GATA3 and IL-4 in peripheral blood CD4^+^ T cells isolated from patients with or without AAD were measured by real-time quantitative polymerase chain reaction (RT-qPCR) and Western blotting, respectively. Finally, Co-immunoprecipitation (Co-IP) was employed to identify the interaction between livin and GATA3.

**Results:**

Compared with WT mouse, Th2 cell frequency in lung tissues and spleen was significantly decreased in livin KO mouse; after adding Lp-15, the differentiation from Naive CD4^+^T cells in spleen to Th2 cells was blocked; the protein and mRNA levels of livin, GATA3 and IL-4 in AAD group were higher than that in control group. The levels of livin were positively correlated with IL-4, and GATA3 was also positively correlated with IL-4 and livin. GATA3 was detected in the protein complex co-precipitated with livin antibody, and livin was also detected in the protein complex co-precipitated by GATA3 antibody.

**Conclusion:**

Livin increases the expression of IL-4 and facilitates naive CD4^+^ T cells to differentiate into Th2 cells, which triggers airway allergy.

## Introduction

Airway allergic diseases (AAD) include allergic rhinitis (AR) and bronchial asthma (asthma) [1, 2]. The World Health Organization (WHO) estimates that about 300 million people worldwide suffer from asthma and 400 million from allergic rhinitis, and more than 180,000 people die of asthma each year, making allergic diseases a global health problem [3, 4]. The Th2-biased immune response is one of the core mechanisms of AAD, but the underlying mechanism is still unclear. In the preliminary studies, by human genome-wide oligonucleotide microarray hybridization and analysis, we found that the expression of the apoptosis suppressor protein livin was significantly high in the nasal mucosa of patients with AAD. The data imply that livin is closely related to the onset of airway allergy and may be involved in the formation of Th2 polarization in AAD [5]. Livin is an inhibitor of apoptosis protein (IAP) family member and can be found in many cancers [6]. However, other researches showed that livin plays an important role in autoimmune and chronic inflammatory diseases, such as psoriasis [7] and chronic rhinosinusitis with nasal polyps(CRSwNP) [8].

Interleukin 4 (IL-4), a signature cytokine of Th2 immune response facilitates naive CD4^+^ T cells to differentiate into Th2 cells[9]. GATA3 is a transcription factor for IL-4[10]. It is reported that the expression level of GATA3 in CD4^+^ T cells of AAD mice was significantly increased compared with that of naïve mice[11], which suggests that GATA3 is involved in the development of airway allergy. Based on information mentioned above, we hypothesize that livin may interact GATA3 to regulate IL-4, and promote the Th2 immune response. This study mainly elaborates the mechanism of livin involved in the pathogenesis of AAD to provide new understanding for the further research of the pathogenesis of airway diseases.

## Materials and methods

### Human subjects

From March 2019 to December 2020, 30 patients with airway allergic disease were recruited into this study at the Second Hospital, Shanxi Medical University. AAD was diagnosed based on our routine procedures by our physicians{inclusion criteria: Guidelines for diagnosis and treatment of allergic rhinitis ( Tianjin, 2015)}. The baseline characteristics of human subjects are presented in Table 1. All patients had stopped local or systemic medication for one month. Participants with one of the following conditions were excluded from the study: cancer; autoimmune diseases; severe organ diseases; or using immune suppressors or anti-cancer medicines. 30 healthy subjects were also recruited as controls. The experimental procedures were approved by the Human Ethics Committee at Second Hospital of Shanxi Medical University (approval date: 201,903). Each human subject gave their written informed consent. There is no significant difference in age, gender and BMI between the two groups (P > 0.05).

**Table 1:** Demographic data of human subjects

Characteristic	Control group	AAD group
Subjects, n	30	30
age (years), mean ± SD	30.5 ± 4.5	31.3 ± 5.3
Gender (female/male), n	18/12	14/16
Body mass index (kg/m2), mean ± SD	25.4 ± 3.7	26.1 ± 4.5
FEV1% predicted, mean ± SD	108 ± 10.1	82.4 ± 11.6 a
FEV1/FVC (%), mean ± SD	85.7 ± 5.4%	71 ± 4.8% a
IgE (IU/mL) b	21(4.8–35.7)	153(65.6–247.6) a
Eosinophil count (per µL) b	82 (66–116)	204 (123–289) a
C-reactive protein (mg/L) b	0.89 (0.42–2.7)	1.6 (0.9–4.5) a

Clinical characteristics and statistics of candidates (± s)

a: P < 0.05 compared with the healthy group (t test); b: The representation of the data: median (lower quartile-upper quartile).

### Mice

The CD4-livin knockout mice (the livin gene was conditionally knocked out in CD4^+^ T cells and other cells expressed livin normally, hereinafter: KO mice) were provided by the Cyagen Biosciences Inc. The wild-type (WT) mice (C57BL/6 mice, 6–8-week-old) were purchased from the Shanxi Medical University Experimental Animal Center. All mice were maintained under specific pathogen-free condition and could access water and food freely. The animal experiments were approved by the Animal Ethical Committee at Shanxi Medical University.

### Reagents

The reagents and materials for real-time qualitative polymerase chain reaction (RT-qPCR), Western blotting, and TRIzol were purchased from Invitrogen (Carlsbad, CA). The immune cell isolation kits were purchased from Miltenyi Biotech (San Diego, CA). The Co-immunoprecipitation (Co-IP) and Immunomagnetic bead sorting kits were purchased from Thermo Fisher Scientific (Cambridge, MA). The reagents for IP Lysis Solution, Protein G agarose and rotational cylindrical were purchased from Shanghai Sangon Biotech (Shanghai, China). Antibodies of livin, IL-4 and GATA3 were purchased from Santa Cruz Biotechnologies (Santa Cruz, CA). Fetal bovine serum was purchased from Gibco. Ficoll lymphocyte isolation fluid was purchased from Sigma(USA). The fluorochrome-labeled antibodies of IL-4 were purchased from Thermo Fisher Scientific. Livin inhibitor was purchased from Calbiochem (Darmstadt, Germany).

### AAD mouse model development

WT mice were sensitized by subcutaneous injection with ovalbumin (OVA, 100 µg/mouse) mixed in 0.1 ml alum on day 1, 3 and 7, respectively. From day 15, mice were treated with nasal instillation (50 µl/nostril) containing OVA (50 μg) mixed in 50μL phosphate-buffered saline (PBS), once a day, 7 times in total. Mice were anesthetized by intraperitoneal injection of 1% sodium pentobarbital (50 mg/kg) on day 22, and abdominal aorta bleeds to death. Lung tissues and spleen were quickly collected for related studies.

### Isolation of mononuclear cells

Preparation of human peripheral blood mononuclear cells: The blood samples were collected from AAD patients and healthy subjects via ulnar vein puncture. Peripheral blood mononuclear cells (PBMCs) were isolated from the samples by density gradient centrifugation.

Isolation of mononuclear cells from lung and spleen of mice: The lung and spleen were collected from mice, cut into small pieces, and digested with digestive solution containing collagenase I and DNAzyme, and then passed through 200 mesh screens. The mononuclear cells were isolated with 1.083 g/ml ficoll lymphocyte isolation solution (sigma, USA).

### Isolation and culture of CD4.^+^ T cells

Following the manufacturer’s instructions, immune cells (CD4^+^ T cells) were isolated from the PBMCs by magnetic cell sorting (MACS) with relevant reagent kits. The cell purity was checked by flow cytometry. MACS was performed again if purity did not reach 95%. Cells were cultured in Roswell Park Memorial Institute1640 (RPMI1640) culture medium containing fetal bovine serum (10%), streptomycin (0.1 mg/mL), penicillin (100 U/mL), and L-glutamine (2 mM). The medium was changed in 2 to 3 days. The cell viability was greater than 99% as assessed by Trypan blue exclusion assay.

### Flow cytometry

Cells were collected from relevant experiments. The cells were incubated for surface staining with relevant antibodies or isotype IgG for 30 min at 4 °C. For intracellular staining, cells were treated with fixative and permeable reagents for 1 h and then incubated with relevant antibodies or isotype IgG for 30 min at 4 °C. The samples were analyzed with a flow cytometer (BD FACSCanto II). The results were acquired and analyzed with a FlowJo software package. Data of isotype IgG staining were used as gating references.

### Real-time quantitative RT-PCR (RT-qPCR)

RNA was extracted from the cells with TRIzol reagents and converted to cDNA with a reverse transcription kit according to the manufacturer’s instruction. The samples were amplified in a qPCR device with the SYBR Green Master Mix and the presence of relevant primers, including livin, GATA3 and IL-4 (Table 2). The results were calculated with the 2-∆∆Ct method and presented as relative quantification (RQ).

**Table 2:** Primer sequences used for RT-qPCR

Gene	sequence
livin	F: 5’-AGGGCGTGGTGGGTTCTTG-3’
	R: 5’-CGGCACAAAGACGATGGACA-3’
GATA3	F: 5’-CCAGGCAAGATGAGAAAGAGTG-3’
	R: 5’-ATAGGGCGGATAGGTGGTAATG-3’
IL-4	F: 5’-TCTCACCTCCCAACTGATCC-3’
	R: 5’-TTGCTGTGAGGATGTTCAGC-3’
GAPDH	F: 5’-TCAAGAAGGTGGTGAAGCAG-3’
	R: 5’-AGGTGGAAGAATGGGAGTTG-3’

F: forward primer; R: reverse primer

### Western blotting

CD4^+^ T cells in peripheral blood were lysed in Laemmli buffer. Lysates were centrifuged at 13,000 xg for 15 min at 4 °C. Supernatant was collected and detected the levels of livin, IL-4 and GATA3 protein. The protein was separated by sodium dodecyl sulphate–polyacrylamide gel electrophoresis (SDS-PAGE) and transferred onto a nitrocellulose membrane. Anti-livin, or IL-4, or GATA3 antibody were incubated with the membrane overnight at 4 °C. Second antibodies (labeled with HRP) were then added, and incubated for 2 h. Immunoblots on the membrane was developed by the enhanced chemiluminescence, and recorded with photographing.

### Co-immunoprecipitation (Co-IP)

The cells were collected and washed with PBS, added the pre cooled IP lysate at 500 μl, incubated on ice for 5 min and mixed regularly. The products of cell lysis were transferred to a centrifuge tube, and centrifuged at 13,000 xg for 10 min to precipitate cell fragments. The supernatant was transferred to a clean centrifuge tube (cell lysate) for relevant operation. A small quantity of lysates was analyzed by Western blotting. Take 1 μg isotype IgG and 30 μl 50% protein G agarose into 200 μL cell lysates to incubate for 30 min at 4 ℃. Samples were centrifuged at 10,000 xg for 1 min. The beads were discarded; the supernatant was collected for subsequent experiment. 4 μg rabbit anti human livin antibody or isotype IgG was added to the centrifuge tube containing the pre-cleared cell lysate and incubated overnight at 4 ℃. 30 μl 50% protein G agarose were added to be incubated for 1 h at 4 ℃, and centrifuged at 10,000 xg for 1 min to remove the supernatant. The beads were washed 3 times. Proteins on the beads were eluted using eluting buffer, and analyzed by Western blotting.

## Statistics

Spss22.0 software was used for statistical analysis. The data were presented as mean ± standard deviation (SD). The difference between 2 groups was determined by Student t test or analysis of variance (ANOVA) if more than two groups. Non-normal distribution with rank and test. Spearman was adopted for correlation analysis. A value of p < 0.05 was set as a significant criterion.

## Results

### The ratio of Th2 cells in airway and spleen in WT mice and KO mice

**Fig. 1:**
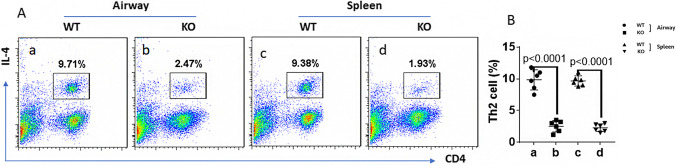
Depletion of livin expression inhibits Th2 cells (IL-4^+^)differentiation. The WT mice and livin KO mice (KO; the mice carrying livin-KO CD4^+^ T cells) were sensitized to OVA (sensitized). **A** Gated flow cytometry plots show Th2 cell counts collected from airway and spleen in WT mice (n = 6) and livin KO mice (n = 6). **B** Summarized data of A. OVA = ovalbumin; KO = knock out; WT = wild-type

Flow cytometry analysis was performed to determine the levels of IL-4 positive cells (Th2 cells) in airway and spleen. The results showed that, compared with WT mice, the Th2 cell frequency in the lung tissues and spleen was decreased significantly in livin KO mice. The difference was statistically significant (p < 0.0001) (Fig. 1).

### The effect of livin inhibitor on the differentiation of naive CD4^+^ T cells in the spleen of WT mice

**Fig. 2:**
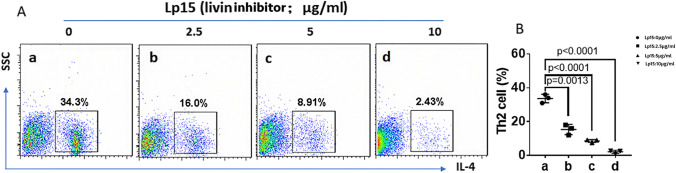
Inhibition of livin blocked Th2 cell differentiation. Naive CD4^+^ T cells were isolated from spleen and cultured in Th2 inducer and livin inhibitor.** A** Gated flow cytometry dot plots show Th2 counts cultured in different potency of livin inhibitor.** B** Scatter plots indicate summarized data of Th2 cells. WT = wild-type

The Th2 inducer【IL-4 (4 ng/ml), anti-IFN-γ (20 μg/ml), anti-IL-12 (20 μg/ml), IL-2 (10 ng/ml), anti-CD3 (6 μg/ml), and anti-CD28 (6 μg/ml)】and livin inhibitor (Lp-15) were added into naive CD4^+^ T cell culture isolated from the spleen of WT mice, and cultured for four days. Flow cytometry was performed to assess the levels of IL-4 positive cells (Th2 cells). The results showed that the Th2 cell frequency in the spleen was significantly decreased after livin inhibition (p < 0.001) (Fig. 2).

### The expression of livin mRNA and IL-4 mRNA in peripheral blood CD4^+^ T cells of AAD group and control group and their correlation.

**Fig. 3:**
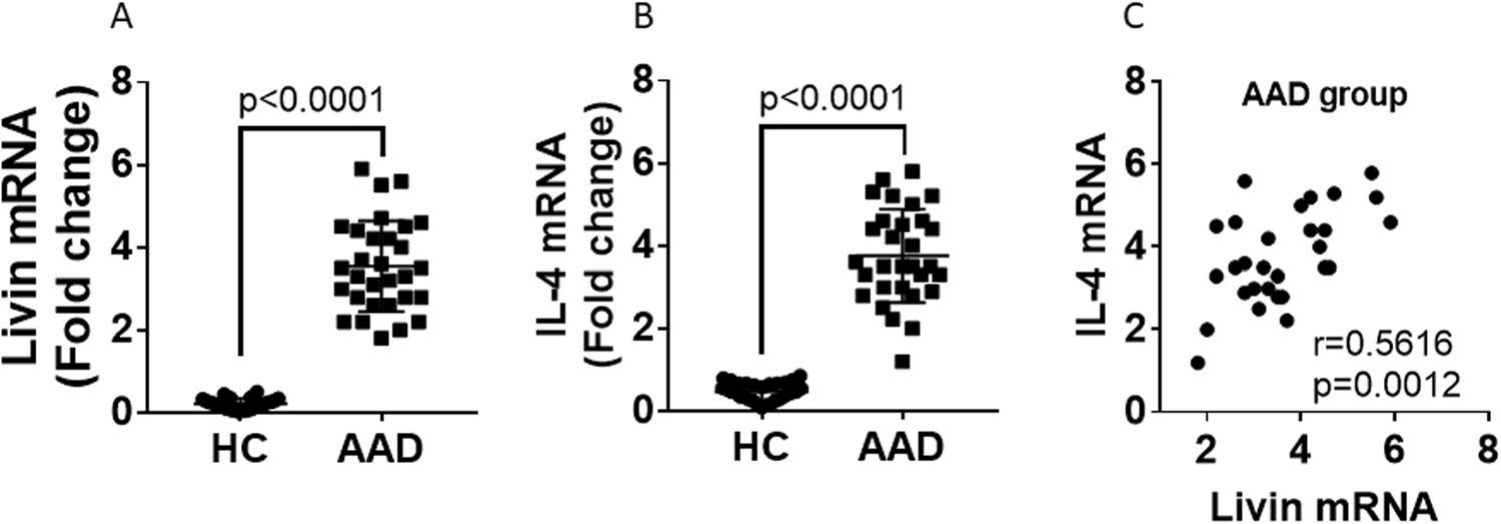
CD4^+^ T cells in AAD patients express high levels of livin mRNA and IL-4 mRNA and livin mRNA are positively correlated with IL-4 mRNA expression. **A, B** The scatter plots indicate the levels of Livin mRNA and IL-4 mRNA in CD4^+^ T cells isolated from blood samples collected from HC (n = 30) and AAD (n = 30). **C** The correlation between the levels of Livin mRNA and IL-4 mRNA of patients with AAD. HC = healthy control; AAD = airway allergic disease

The mRNA expression of livin and IL-4 was assessed by RT-qPCR in peripheral blood CD4^+^ T cells. The expression levels of livin mRNA and IL-4 mRNA in AAD patient group were higher than that in the healthy control group (P < 0.0001) (Fig. 3A, 3B). Spearman correlation analysis showed that the expression of livin mRNA was positively related to IL-4 mRNA (r = 0.5616, p = 0.0012) (Fig. 3C).

### The expression of livin and IL-4 protein in peripheral blood CD4^+^ T cells of AAD group and control group.

**Fig. 4:**
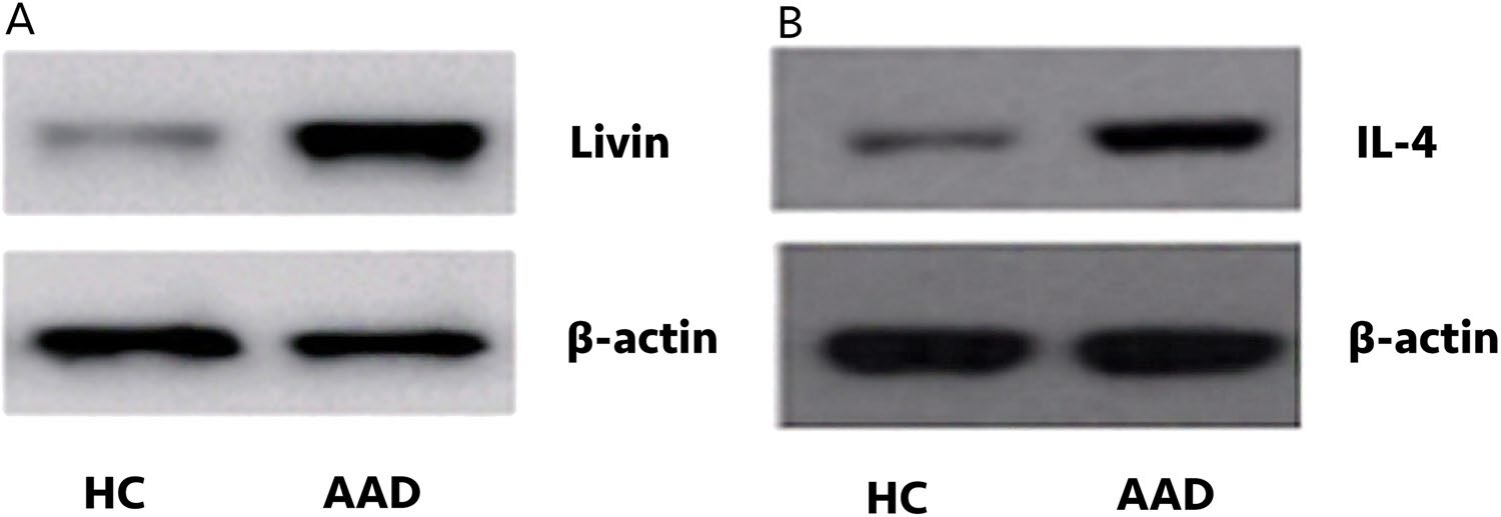
CD4^+^ T cells in AAD patients express high levels of livin and IL-4. The blood samples were collected from AAD patients (n = 30) and HC (n = 30) and the CD4^+^ T cells were isolated from the blood samples by MACS. The immunoblots show livin (**A**) and IL-4 (**B**) protein levels in CD4^+^ T cells. HC = healthy control; AAD = airway allergic disease; MACS = magnetic cell sorting

The expression of livin and IL-4 protein was assessed by Western blotting in peripheral blood CD4^+^ T cells. The expression level of livin and IL-4 protein in AAD group was higher than that in the control group (P < 0.0001) (Fig. 4).

### The expression of GATA3 mRNA and GATA3 protein in peripheral blood CD4^+^ T cells of AAD group and control group, and the correlation between GATA3 mRNA and IL-4 mRNA or livin mRNA, and the interaction of livin and GATA3.

**Fig. 5:**
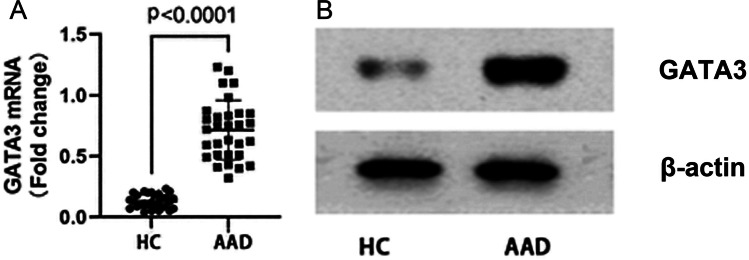
CD4^+^ T cells in AAD patients express high levels of GATA3 mRNA and GATA3. CD4^+^ T cells were isolated from PBMCs of AAD patients (n = 30) and HC (n = 30). **A** The scatter plots show the levels of GATA3 mRNA in CD4^+^ T cells. **B** The immunoblots show the levels of GATA3 protein in CD4^+^ T cells. PBMCs = peripheral blood mononuclear cells; HC = healthy control; AAD = airway allergic disease

**Fig. 6:**
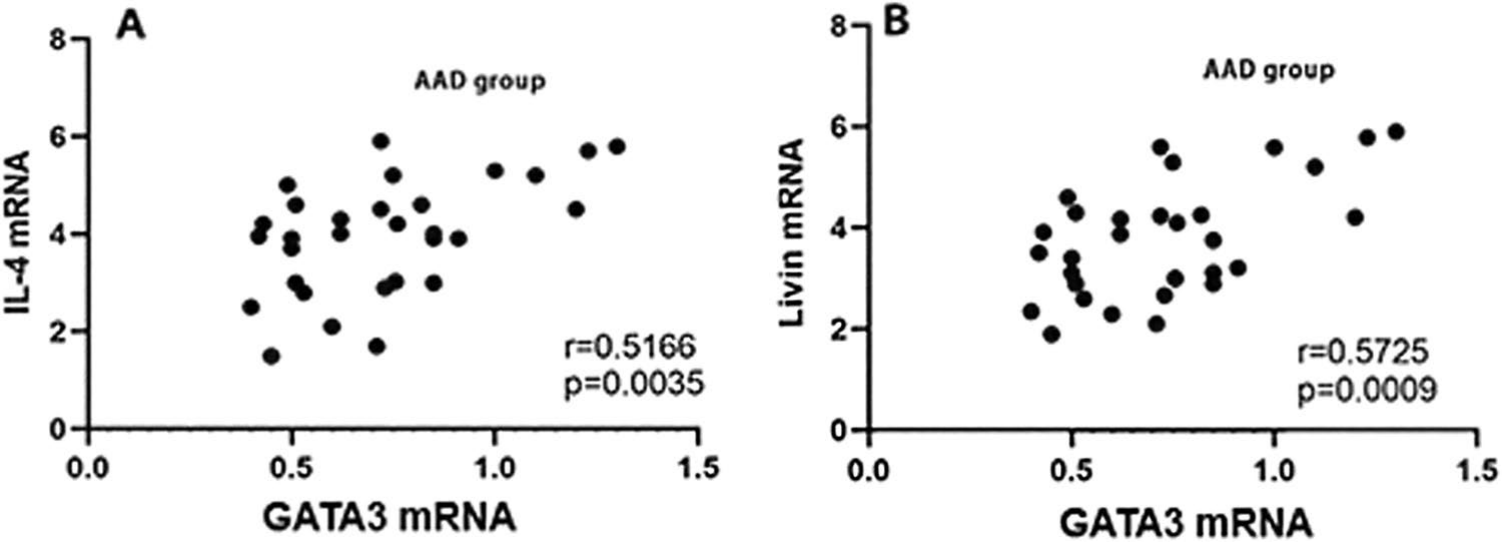
GATA3 mRNA are positively correlated with IL-4 mRNA and livin mRNA levels in AAD group. **A** The scatter plots show the correlation between the mRNA levels of GATA3 and IL-4 of AAD subjects (n = 30). **B** The scatter plots show the correlation between the mRNA levels of GATA3 and livin of patients with AAD (n = 30). AAD = airway allergic disease

**Fig. 7:**
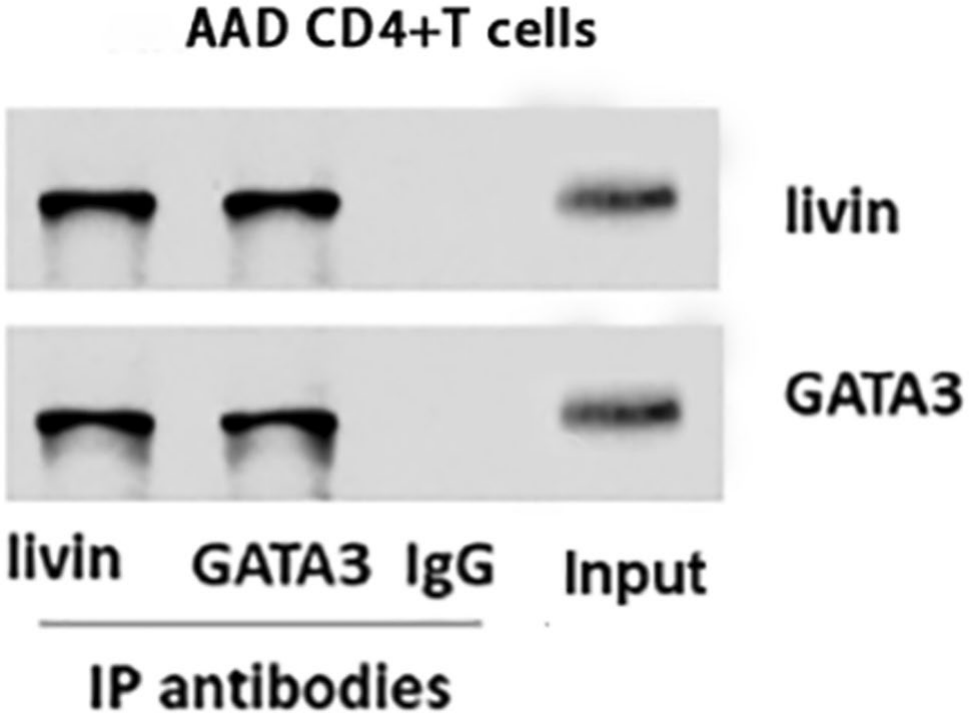
Livin and GATA3 form a complex in CD4^+^ T cells. CD4^+^ T cells were isolated from peripheral blood samples of AAD patients. Proteins were extracted from 30 AAD patients and analyzed by Co-IP. The results were verified by Co-IP with either anti-livin antibody (Ab) or anti-GATA3 Ab as precipitating Abs. Immunoblots show a complex of livin and GATA3 in the CD4^+^ T cells of AAD. AAD = airway allergic disease; Co-IP = Co-immunoprecipitation

RT-qPCR and Western blotting were performed to measure the expression level of GATA3 mRNA and protein. The levels of GATA3 mRNA and protein in AAD group were higher than that in the control group (p < 0.05) (Fig. 5). Spearman correlation analysis showed that the expression of GATA3 mRNA was positively related to that of IL-4 mRNA and livin mRNA (r = 0.5166, p = 0.0035; r = 0.5725, p = 0.0009)(Fig. 6). The protein was extracted and analyzed by immunoprecipitation. The results of Western blotting were obtained by chemiluminescence and imaging. The result showed that livin and GATA3 interaction to form a complex in peripheral blood CD4^+^ T cells in the AAD group (Fig. 7).

## Discussion

The incidence of allergic airway diseases has exceeded 10% in the world and showed a rising trend year by year [12, 13], which brings a huge burden to human health and social economy [14]. Previous studies showed that AAD is an allergic inflammation of respiratory mucosa characterized by Th2 polarization [15]. Our previous study suggested that livin is closely related to the pathogenesis of AAD. In the present study, we used livin KO mice to observe the effect of livin on Th2 differentiation, and found that the Th2 cell frequency in the lung tissues and spleen of livin KO mice was significantly lower than WT mice. Meanwhile, livin inhibitor (Lp-15) was added to the naive CD4^+^ T cells culture medium isolated from the spleen of WT mice to inhibit the expression of livin. With the increase of livin inhibitor concentration, the Th2 cell frequency was decreased. It indicates that livin inhibitor can block the differentiation of Th2 cells, and livin plays a key role in Th2 differentiation.

Interleukin 4 (IL-4) is the signature cytokine of T helper 2 (Th2) response. During the differentiation of Th2 cells, IL-4 is produced by Th2 cells. Through paracrine or autocrine manner, IL-4 not only stimulates the non-producing cells to produce IL-4, but also enhances the ability of IL-4 producing cells to produce IL-4 [16, 17]. Overproduction of IL-4 plays a critical role in the pathogenesis of airway allergy. Many studies have tried to regulate the production of IL-4 to suppress airway allergy disorders. However, the therapeutic efficacy of remedies in the regulation of IL-4 production is still unsatisfactory. GATA3 is a transcription factor of IL-4 [18], which plays an important role in promoting the differentiation of Th2 cells and inducing the production of cytokines [19, 20]_._ Previous clinical trial reported that using a DNA enzyme in AAD patients can inactivate GATA3 mRNA and improve the clinical symptoms of AAD [21], which indicates that GATA3 plays an important role in the pathogenesis of AAD. In our study, the expression of livin, GATA3 and IL-4 in peripheral blood CD4^+^ T cells of the two groups was detected by RT-qPCR and Western blotting. It was found that livin, GATA3 and IL-4 expressed in both control group and AAD group, and the expression of livin, IL-4 and GATA3 in the AAD group were significantly higher than the control group. This result is corresponding to previous studies, that is, the expression of GATA3 in CD4 ^+^ T cells of AAD patients was significantly higher than that of health person [11]. Meanwhile, IL-4 is expressed highly in peripheral blood CD4 ^+^ T cells of AAD. The expression of IL-4 is positively correlated with the expression of livin and GATA3 and livin is also positively correlated with GATA3. It is suggested that the high expression of livin may enhance the expression of IL-4; moreover, GATA3 participates the synthesis of IL-4 by stimulating its express [22] and, then, promotes the differentiation of Th2 cells.

Livin is a member of inhibitor of apoptosis protein family. It can inhibit apoptosis-related proteins such as caspase 3, 7 and 9 for anti-apoptotic effects [23]. In addition, livin can also induce the production of specific CD4^+^ T cells and CD8^+^ T cells [14]. Most of the previous studies on livin focused on the pathogenesis of tumor, such as the relationship between livin expression and the pathogenesis and prognosis of colon cancer and nasopharyngeal carcinoma [24, 25]. Recent reports depicted that stronger expression of livin in the pathogenesis of chronic rhinosinusitis with nasal polyps (CRSwNP). Furthermore, Caspase-3 and second mitochondria-derived activator of caspases (Smac) expression were suppressed by livin for exerting its anti-apoptosis effect, especially in eosinophilic CRSwNP (ECRSwNP) [8]. Interestingly, Xue et al. newly discovered that livin played a critical role in ECRSwNP. Livin facilities nasal chronic inflammation by promoting the thymic stromal lymphopoietin (TSLP) expression in nasal epithelial cells [26]. Apparently, it is different with conclusion as described previously. It can be concluded that livin presents various pathogenic roles in chronic airway inflammatory disease. To date, there is little information on livin in the pathogenesis of airway allergic diseases. In previous studies, our group used lentivirus-mediated RNAi and co-immunoprecipitation had clarified survivin had positive regulation on the expression of IL-4 in CD4 ^+^ T cells [27, 28], and confirmed that survivin plays a critical role in the pathogenesis of airway allergy. But what is the mechanism of anti-apoptotic livin to regulate the expression of IL-4 and promote the immune response of Th2 cells?Co-immunoprecipitation confirmed that livin interacted with GATA3 and formed a protein complex. We believe that the complex formations of livin/GATA3 enhances the stability of livin expression that triggers the high expression of IL-4 by activating the transcription of IL4 gene and facilitates CD4^+^ T cells to differentiate into Th2 cells, and causes airway allergy.

However, the co-binding region and functional targets of livin / GATA3 have not been identified. We may further use ChIP to identify its binding sites and targets, and observe the characteristics of livin/GATA3 binding peak combined with high-throughput sequencing (ChIP-seq), so as to clarify the transcription regulation mechanism of livin/GATA3 complex. Additionally, we may observe the regulatory effects of livin on GATA3 expression and the expression of Th2 cytokines by the livin gene knockout experiments.

In summary, the data provide a new idea for further research of the pathogenesis of airway diseases, and potential clinical drug targets through the study of the mechanism of livin promoting Th2 differentiation in allergic airway diseases.
